# Novel Compounds for Hair Repair: Chemical Characterization and In Vitro Analysis of Thiol Cross-Linking Agents

**DOI:** 10.3390/ph18050632

**Published:** 2025-04-27

**Authors:** Sami El Khatib, Dalal Hammoudi Halat, Sanaa Khaled, Ahmed Malki, Bassam Alameddine

**Affiliations:** 1 Department of Biomedical Sciences, School of Arts and Sciences, Lebanese International University, Bekaa P.O. Box 146404, Lebanon; 2 Center for Applied Mathematics and Bioinformatics, Gulf University for Science and Technology (GUST), West Mishref 32093, Kuwait; 3 QU Health Office of Assessment and Accreditation, QU Health, Qatar University, Doha P.O. Box 2713, Qatar; dhammoude@qu.edu.qa; 4 Department of Chemical Sciences, School of Arts and Sciences, Lebanese International University, Bekaa P.O. Box 146404, Lebanon; sanaa.khaled@liu.edu.lb; 5 Department of Biomedical Sciences, College of Health Sciences, QU Health, Qatar University, Doha P.O. Box 2713, Qatar; ahmed.malki@qu.edu.qa; 6 Functional Materials Group, Gulf University for Science and Technology, Mubarak Al-Abdullah 32093, Kuwait; alameddine.b@gust.edu.kw

**Keywords:** hair damage repair, disulfide bridge regeneration, thiol-reactive cross-linking agents, tensile strength test, cysteine reactivity test, in vitro hair treatment evaluation

## Abstract

**Introduction:** Hair damage from chemical treatments, mechanical stress, and environmental factors can lead to significant degradation in hair quality, necessitating effective solutions for restoration. The aim of this study was to develop and evaluate novel compounds for repairing hair damage through the chemical regeneration of disulfide bridges. **Materials and Methods:** Three novel thiol-reactive cross-linking agents (APA, STA, SAA) were synthesized and characterized. Their efficacy in repairing hair damage was evaluated through in vitro tensile strength tests on human hair fibers, comparing treated and untreated samples. Cysteine reactivity tests were also performed to assess the capability of these agents to restore disulfide bridges in hair keratin. **Results:** The tensile strength tests revealed significant improvements in the mechanical properties of treated hair fibers compared to untreated samples. APA demonstrated the highest efficacy in restoring tensile strength and elasticity, showing higher performance in mechanical strengthening. The cysteine reactivity tests confirmed that APA could effectively re-establish disulfide bonds, particularly at higher temperatures. STA, while less effective than APA, showed substantial efficiency in restoring disulfide bonds. When compared to the reference agent, both APA and STA exhibited higher performance in tensile strength and cysteine reactivity, with APA showing the greatest improvement in mechanical properties. **Conclusions:** Our study successfully revealed the potential of the synthesized thiol-reactive cross-linking agents in repairing hair damage by chemically regenerating disulfide bridges. These findings offer a promising new direction for the development of advanced hair repair treatments in the cosmetic industry.

## 1. Introduction

One of the most symbolic elements of our physical identity, human hair serves as a biological protection to the scalp, as well as an authentic expression of an individual’s appearance. Damage, graying, aging, dandruff, alopecia, and other conditions affecting hair can be quite distressing [1]. As such, the growing influence of the global cosmetic industry and current dermatological practice has led to an increased focus on addressing the problem of hair damage within the realms of dermatology and cosmetology. This emphasis is driven by the need to meet consumer expectations for maintaining the aesthetic appeal and quality of hair [2]. Currently, hair care and lifestyle play important roles in overall physical appearance and self-perception for both men and women [3]. As such, the hair care industry is particularly focused on creating and advancing products designed to sustain the appearance of healthy hair. This emphasis underscores the extensive global market size of the hair care industry, which is a multibillion-dollar enterprise worldwide [4].

Normally, hairs are complex, multilayered, protein filaments that grow from follicles located in the dermal skin, with each hair consisting of two distinct parts: the deep follicle or bulb located in the dermis, which includes stem cells for hair regeneration, and the shaft, the hard, filamentous structure extending above the skin surface [5]. A cross-section of the shaft, whose average thickness ranges from 60 to 80 μm, shows three distinct layers: the cuticle, the medulla, and the cortex [6,7]. The cuticle is the outermost protective structure that forms a shield of several layers of flat, thin, superimposed scales that cover the surface of the hair, with an overlapping arrangement often compared to “roof shingles”. It acts as a barrier, protecting the central cortex, which houses the innermost medulla, the latter being a loosely packed, disorganized, central area surrounded by the cortex [8]. The hair cortex is the most voluminous part of the hair fiber and consists of spindle-shaped cells that lie parallel to the hair axis [9]. Approximately 90–95% of these cortical cells are composed of the helical protein keratin, longitudinally arrayed in the form of intermediate filaments (IFs) along with keratin-associated proteins (KAPs) [10,11], which are crucial for hair health, tensile strength, and rigidity via the formation of a cross-linking network with IFs [9,12]. At the molecular level, structural hair damage involves the disruption of disulfide bonds (–S–S–) between cysteine residues, which are critical for covalent cross-linking and mechanical stability of the keratin network. In addition, hydrogen bonds, ionic interactions, and hydrophobic forces that maintain keratin’s secondary and tertiary structures are also affected by chemical and thermal stress [13].

Keratin, the principal structural protein in hair, is composed of α-helical polypeptide chains arranged into coiled-coil motifs that form intermediate filaments [14,15]. These filaments are stabilized by covalent disulfide cross-links between cysteine residues, which contribute to the mechanical strength, elasticity, and chemical resistance of the hair fiber [16]. These chains can either curl into helices, giving rise to an α-conformation, or can bond side-by-side into pleated sheets, forming the β-conformation, resulting in structurally distinct α- and β-keratins. While β-keratin is recognized as the tougher protein, α-keratin is commonly found in mammals and is the primary constituent of hair [17,18].

Keratin, which is synthesized by keratinocytes of the skin, is a robust, highly stable protein that remains insoluble in water and most organic solvents. It is resistant to high temperature and enzymatic degradation by proteolytic enzymes and exhibits considerable mechanical and chemical resistance [19]. Chemically, keratin is composed of 18 amino acids, the most abundant being cysteine, cystine, serine, glutamic acid, glycine, threonine, arginine, valine, leucine, and isoleucine. Cysteine, in particular, is abundant in keratin, contributing to the formation of disulfide bridges that give hair its mechanical strength and elasticity. The precise configuration of keratin and the presence of these disulfide bonds are both essential for maintaining the strength and structure of the hair [10,20,21]. While cysteine residues essentially help to stabilize the overall assembly of keratins and KAPs, a proportion of their intermolecular disulfide bonds are assumed to be associated with the mechanical flexibility of hair [22]. Notably, the keratin backbone, with inter- and intramolecular bonding and numerous disulfide, hydrogen, and ionic bonds, provides increased stability to the keratin structure, contributing to hair strength [23]. Among these bonds, disulfide bridges appear to be vital for hair strength and toughness [24], as they impart a hydrophobic surface to hair, which is related to their insolubilization properties [25]. The oxidative cleavage of disulfide bridges results in fragile hair [26], and the removal of hydrophobic disulfides results in extreme damage to the hair, structural cracks, and lifting of the cuticle [27].

Despite such chemically and structurally sophisticated architecture, human hair is constantly exposed to harmful agents that can alter both its appearance and tensile properties. Common practices that induce mechanical damage to hair, such as excessive grooming, styling, and perming, or chemical damage, such as coloring, bleaching, and various chemical treatments, all impose considerable stress on the hair structure and alter its wetting characteristics, water retention, combing properties, and mechanical robustness [28]. Moreover, hair dryers can denature hair shaft proteins, imparting hair damage manifested as roughness, dryness, and loss of hair color, especially at high temperatures, prolonged drying times, and drying distances less than 15 cm [29,30]. Environmental factors also take their toll on human hair; prolonged exposure to sunlight is implicated in hair damage. Through a free radical chain oxidation reaction initiated by high-energy UV light propagating in the presence of atmospheric oxygen, hair is ultimately susceptible to brittleness, color changes, loss of luster, split ends, and increased surface friction [31]. Smoking and dietary factors, including malnutrition of essential fatty acids, minerals, and vitamins, whether caused by inborn errors or reduced uptake, are additional factors that impair hair growth and cause damage to existing hair [1,32]. Furthermore, hair anatomy is not constant as we grow, and changes in hair anatomy are a part of the physiological process of aging [33]. As individuals age, the hair shaft diameter tends to diminish, increasing the risk of breakage and resulting in reduced hair shine, dull appearance, and split ends. Additionally, alterations in frictional properties with age increase the difficulty of brushing and grooming the hair, contributing to cumulative aspects of hair damage [34]. As such, all the aforementioned aggressions on hair can lead to harm ranging from dryness and fragility to deeper molecular alterations [18,26,35]. At the molecular level, structural hair damage becomes evident following the destruction of the keratin configuration and the fundamental chemical bonds. Intermolecular ionic, disulfide, and hydrogen bonds, which ensure cohesion between hair proteins, are particularly vulnerable to these aggressions. The rupture of these bonds can lead to loss of strength, decrease in elasticity, and alteration in the texture of the hair [36,37,38,39].

Faced with these challenges, prominent endeavors in hair cosmetology were constantly dedicated to formulating molecules aimed at the restoration of hair structure and containing vital bioactive molecules to minimize, mitigate, or repair hair damage. For instance, preventing hair damage was attempted using nanostructured lipid carriers loaded with vitamin E, formulated as a protective cream [40]. Recently, it was shown that keratin peptides applied to hair samples in vitro can restore moisture and repair chemical bonds, enhancing the mechanical properties of hair [41]. Furthermore, it was found that the use of cysteine with nontoxic polycarboxylic acids could establish bonds between different chemical groups in keratin, increasing intermolecular forces and repairing mechanical damage, suggesting that this combination could replace commercially available perming chemicals that are harmful to hair [39]. Some biotechnology innovations utilizing microRNAs that are important for regulating gene expression in hair follicles have also been leveraged in animal models to stimulate the gene expression needed for growth and hair fiber formation [42,43]. Nevertheless, restoring disulfide bridges by linking broken structures together is an interesting biochemical pathway for reinforcing the stability of keratin and has commercially emerged as a key strategy for improving the quality, strength, and texture of damaged hair [44]. Thus, the need to develop innovative molecules with such a mechanism and incorporate them as active ingredients in a novel class of hair repair formulations remains imperative to the cosmetic industry. The objective of the current study was to perform an in vitro analysis of a set of newly synthesized molecules designed for the purpose of hair damage repair through chemical regeneration of bonds, which replace the damaged disulfide bridges in altered hair samples.

## 2. Results

In addition to ^1^H-NMR, representative ^13^C-NMR spectra were recorded to confirm the structural integrity of carbon backbones in all three salts.

**Scheme 1:**
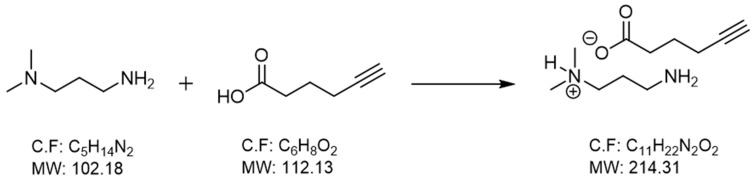
Synthetic route of APA.

**Figure 1:**
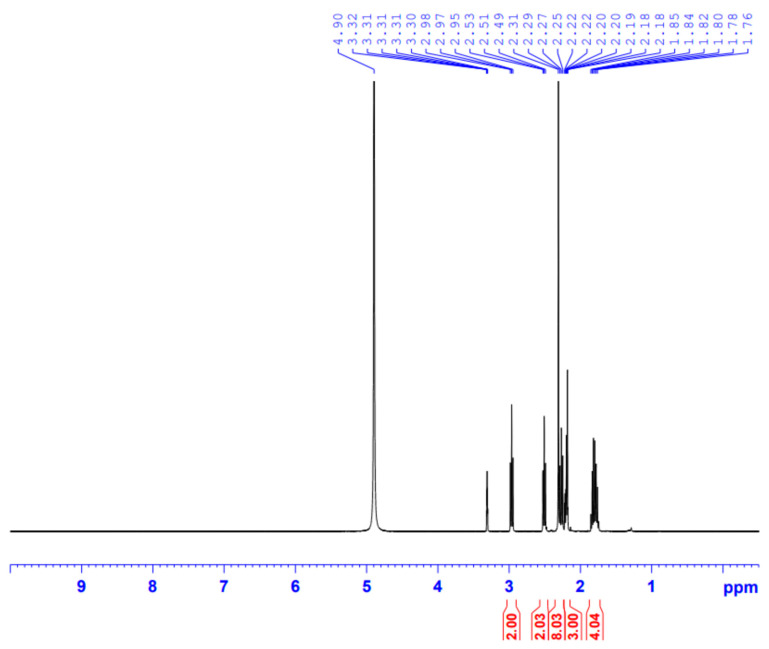
^1^H-NMR spectrum of APA.

All solutions were subjected to a visual appearance test, evaluating color, clarity, homogeneity, and absence of particulate matter or foaming under controlled lighting. Qualified batches appeared colorless to pale yellow, fully transparent, and stable. The appearance test for substance APA revealed a slightly yellow liquid. The obtained water solution was a yellowish solution (2.02 × 10^3^ g) with 98.5% purity and a yield of 97.0% (Scheme 1). Structural verification was performed using ^1^H-NMR and qNMR as primary techniques, complemented by ^13^C-NMR and high-resolution mass spectrometry (HRMS) to provide orthogonal confirmation of molecular structure and molecular weight. While X-ray crystallography was not feasible due to the non-crystalline nature of the salts, spectral data across methods confirmed consistency with the proposed structures. GC analysis confirmed that 98.5% of the product had a total impurity content of 1.3%. The reactivity of APA, STA, and SAA was evaluated relative to the structural and chromatographic behavior of N-Cbz-L-cystine (≥98%, Bachem AG), which served as the reference standard in LC-MS-based adduct profiling assays (see Figure 1).

The synthesis of STA was performed as outlined in Table 1, involving stepwise neutralization and salt formation under ambient conditions. Key process parameters included pH control, controlled amine addition, and post-reaction stabilization. The final STA solution was homogenous, transparent, and pH-stable, with QC confirmation via TLC and visual inspection.

**Figure 2:**
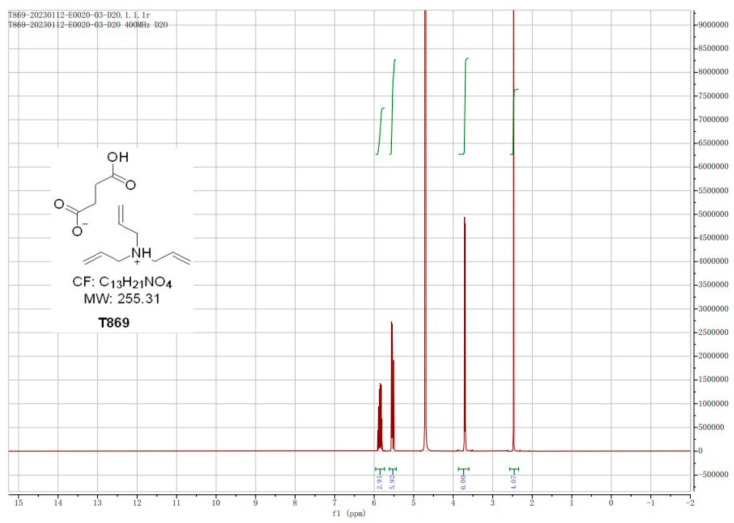
^1^H-NMR spectrum of STA.

The appearance test for substance STA revealed a slightly yellow liquid. HNMR confirmed the presence of a conformable structure (see Figure 2). GC analysis confirmed that 98.5% of the product had a total impurity content of 1.3%. The QNMR results showed that STA conforms to the standard.

**Scheme 2:**
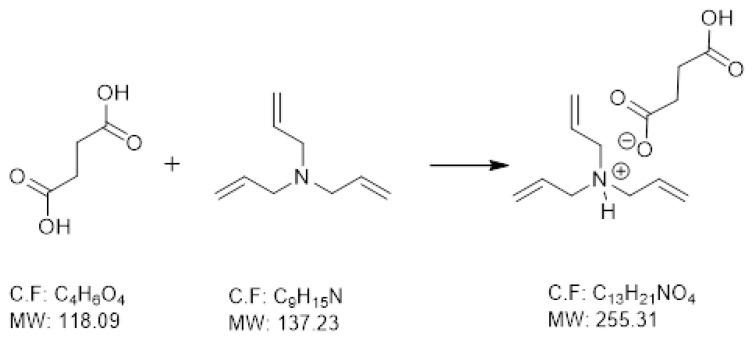
Synthetic route of STA.

**Scheme 3:**
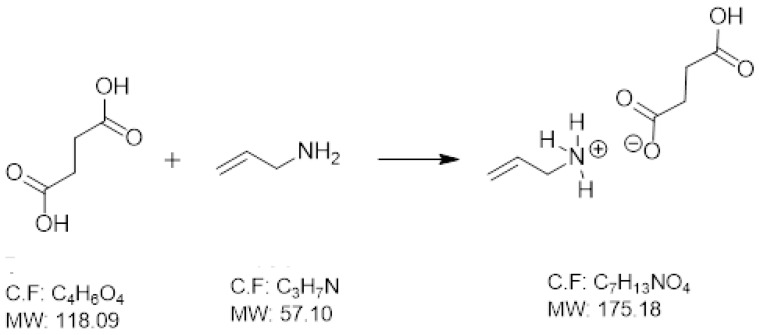
Synthetic route of SAA.

The appearance test of SAA showed that 3.25 × 10^3^ g of a water solution was a colorless solution, and the yield was 96.6% (Scheme 2 and Scheme 3). The HNMR results showed the following: for the SAA-20230300-E0001 batch, the SAA-2 solution was pretreated with NaOH, and then the SAA-1 solution was added dropwise, followed by stirring at room temperature for 3–4 h. The obtained compounds were verified to be correct by HNMR. For the SAA-20230300-E0002 batch, the SAA-2 solution was pretreated with NaOH, extracted with DCM, added dropwise to the SAA-1 acetonitrile solution, and stirred at 65~75 °C for 2–3 h. The resulting white solid was not the desired compound according to HNMR. For the SAA-20230300-E0003 batch, following the reaction conditions of SAA-20230300-E0001, 3.25 kg of the qualified SAA aq. (34.8%) was obtained from 705 g of SAA-1 (Scheme 2 and Scheme 3).

**Figure 3:**
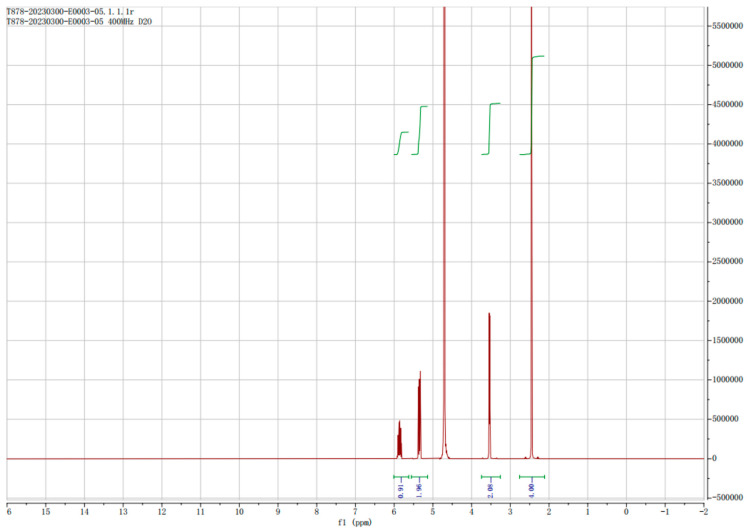
^1^H-NMR spectrum of SAA (succinic acid–allylamine salt) in D_2_O at 298 K, recorded on a 400 MHz spectrometer.

An examination of the appearance of substance SAA revealed that it was a colorless liquid. HNMR confirmed the presence of a conformable structure (see Figure 3). GC analysis confirmed that 97.9% of the product had a total impurity content of 1.3%. The QNMR results showed that SAA conforms to the standard.

The results of the cysteine reactivity test showed that only mono-adducts were present in APA and STA after 30 min at 30 °C, and no bis-adducts were detected by liquid chromatography–mass spectrometry (LC–MS). For SAA, no mono was detected in the reaction mixture at 30 °C, but when the reaction proceeded at 40 °C for 1 h, the mono-adducts were detected by LC–MS. Then, cysteine reactivity was tested at 50 °C while using APA/STA/SAA as the active ingredient, and LCMS revealed that ~12% of the mono- and bis-adductions were present in the APA buffer mixture after 1 h; however, for STA and SAA, no bis-adductions were detected in the buffer mixture after 1 h.

Reactivity toward thiol-containing substrates was quantified via LC-MS following incubation with N-Cbz-L-cystine (see Materials and Methods, Cysteine Reactivity Assay and LC-MS Analysis). The composition of each test compound was confirmed by Elemental Analysis (EA), performed on freeze-dried samples using a Vario EL Cube CHN analyzer. The measured C/H/N ratios matched theoretical values within ±0.4%, supporting the assigned molecular structures. HNMR confirmed that the obtained structure was correct, but some EA was present in the product. LCMS revealed mono- and bis-adductions in APA, mono- and bis-adductions in STA, and mono-adductions in SAA.

When increasing the N-Cbz-L-cysteine to (4.0 eq.) at pH = 5.4 buffer (10 V), the cysteine reactivity of APA, according to LCMS, showed that APA was the only compound to form both mono-thiol adducts (one cysteine–compound linkage) and bis-thiol adducts (two linkages involving both cysteine thiols), confirming its bifunctional thiol-reactivity. In contrast, STA and SAA formed only mono-adducts under the same conditions. Under the same conditions, LCMS for STA showed that the ratio of mono/bis was 41.03%:0% in the reaction mixture after 0.5 h, according to LCMS, and the ratio was 47.24%:0% after 1 h. However, for SAA, LCMS showed that no mono-adducts were produced in the reaction mixture at 30 °C after 1 h, according to LCMS; however, when the temperature was increased to 40 °C, 26.99% of the mono-adducts were generated after 1 h.

As shown in Table 2, APA exhibited temperature-dependent bis-adduct formation, reaching 81.5% at 50 °C. In contrast, STA and SAA produced only mono-adducts under all conditions, confirming their single-point thiol reactivity. These findings support APA’s enhanced cross-linking potential.

When the temperature was increased to 50 °C under the same conditions, the cysteine reactivity of APA and N-Cbz-L-cysteine (4.0 eq.) in pH = 5.4 buffer (10 V) at 50 °C was measured; LCMS showed that the ratio of mono/bis was 17.41%:76.72% in the reaction mixture after 0.5 h, and the ratio was 11.99%:81.54% after 1 h. For STA, LCMS showed that the ratio of mono/bis was 96.81%:0% in the reaction mixture after 0.5 h, and the ratio was 97.79%:0% after 1 h. For SAA, LCMS showed that the ratio of mono/bis was 38.55%:0% in the reaction mixture after 0.5 h, and that the ratio was 73.20%:0% after 1 h.

For the results of the tensile test, before data analysis, the measuring curves were checked for peculiar curve progression that could occur, for example, if the hair fiber was broken or was pulled out of the crimps holding the hair fiber in place. Values resulting from peculiar curve progression were discarded in the following data analysis. Therefore, fewer than 50 results per product were used. Initial testing exhibited a high discard rate due to mounting-related artifacts; however, the methodology was revised to include a pre-tensioning step and improved clamp design. Retesting yielded a discard rate below 15%, and results remained consistent with the original trends. The revised tensile strength data are presented in Table 2 and Figure 1. A mean result of at least 25 hair fibers was regarded as valid. The mean values of the tensile strength at 15% extension, as well as the standard deviations, medians, and differences relative to the baseline, are presented for the treatments in Group 1 and Group 2. A decrease in the tensile strength of 15% is attributed to a positive effect of the products on the tensile strength of the investigated hair.

Comparisons were performed for the changes in the parameters e-modulus and tensile strength of 15% before and after the treatment to a benchmark of 0% between the products within each group (two different formulations, one Olaplex 0-like) and between products with equal concentrations.

Group 1 included hair strands treated immediately after bleaching to model rapid repair intervention, while Group 2 received treatment 24 h after bleaching, simulating delayed or at-home treatment scenarios. The same application protocol and compound concentrations were used in both groups.

Group 1: In comparison to measurements before product application, a significant decrease in the e-modulus tensile strength of 15% after product application was detected for all treatments compared to the benchmark value of 0%, indicating an improved hair status for all treatments. Comparing the treatments to reference Group 1, STA and SAA achieved the best effect.

Group 2: In comparison to measurements before product application, a significant decrease in the parameters e-modulus and tensile strength of 15% after product application was detected for all treatments compared to the benchmark of 0%, indicating an improved hair status for all treatments.

The SAA formulation (previously labeled internally as ‘Reference Code E’) demonstrated moderate recovery of tensile strength, consistent with its mono-thiol reactivity profile. Comparing the treatments to reference code E, no significant differences were found for the parameters e-modulus and tensile strength of 15%. On a descriptive level, APA, compared to Olaplex 0-like 2.3%, achieved the best effect among the products tested in this group.

**Figure 4:**
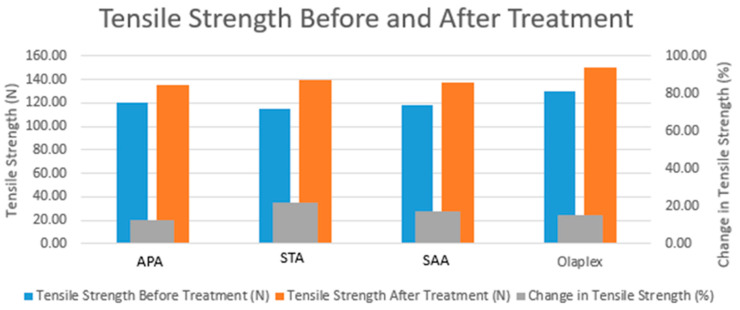
Comparative tensile strength of treated hair fibers. Reference #2 corresponds to Olaplex No. 0. Reference #1 (vehicle control) showed no significant effect.

According to the pairwise comparisons of two different formulations with the same active concentrations, all formulations of Group 1 had greater effects on improving the hair quality than the Olaplex 0-like formulations of Group 2. A significant improvement in hair quality was found for all active substances as well as for both references. Each compound (APA, STA, SAA) was applied at a final concentration of 2.5% *w*/*w*, standardized across all samples to match the reference product (Olaplex No. 0). Stock solutions were diluted accordingly to achieve this concentration, and 100 µL was applied to each strand for 2 min before rinsing. No difference from the reference treatment was detected for any of the Olaplex 0-like formulations in Group 2. Comparing the same concentrations over the two groups, Group 1 showed a better effect on the hair than Group 2, also comparing the two references. Although two reference formulations were described in Section 2, only Reference #2 (Olaplex) is shown in Figure 4 for clarity. Reference #1 (vehicle-only control) showed no significant difference from the untreated group and is therefore omitted from the graphical display.

## 3. Discussion

Our work focuses on repairing hair damage by restoring disulfide bridges through chemical regeneration. This approach is in line with previous studies that have emphasized the importance of disulfide bridges in maintaining hair strength and structure [24,45]. Our manuscript introduces novel molecules designed to replace damaged disulfide bridges, which is a significant advancement in the field of hair repair.

Previous studies have explored the use of bioactive molecules, such as keratin peptides and cysteine, to repair hair damage and enhance hair properties [41,46]. The manuscript further contributes to this area by investigating the biochemical characterization and the repairing potential of three different molecules that can establish bridges between different chemical groups in keratin, thereby affecting the hair strength and texture, and repairing induced mechanical damage. This aligns with previous studies that have investigated the mechanical properties of hair, such as tensile strength and e-modulus, to assess the impact of different treatments on hair quality [26,46]. By quantitatively measuring parameters like tensile strength and e-modulus, the manuscript offers valuable insights into the efficacy of the novel molecules in repairing hair damage. Our manuscript also includes a detailed analysis comparing the effects of different formulations and concentrations on hair quality. Such an approach is consistent with previous studies that have compared the efficacy of various hair care products and treatments in repairing hair damage and improving hair health [28,40].

The use of HNMR and QNMR in the synthesis and characterization of the thiol-reactive cross-linking agents APA, STA, and SAA was pivotal for confirming the chemical structures and purity of these compounds. HNMR spectroscopy provides detailed information about the molecular structure by identifying the types and environments of hydrogen atoms within the molecules [24]. In this study, HNMR was employed to confirm the final products of each synthesized compound, ensuring the correct structures were obtained (see Figure 1, Figure 2 and Figure 3).

QNMR further complements the HNMR by quantifying the exact amounts of the compounds and their impurities [21,45]. This quantitative aspect is vital for assessing the purity and concentration of the synthesized agents, which directly impacts their effectiveness in subsequent experiments. For instance, the results from QNMR indicated that APA had a purity of 98.5%, STA had 92.3%, and SAA had 96.6%, all of which are within acceptable ranges for experimental applications. The high purity levels ensured that the results obtained from subsequent tests were due to the intended compounds and not influenced by significant impurities.

Furthermore, gas chromatography coupled with flame ionization detection was used to analyze the presence of related substances or impurities in the synthesized compounds [41]. This technique is particularly useful for detecting volatile and semi-volatile organic compounds. The use of GC-FID allowed for the separation and quantification of different components within the sample, providing an additional layer of validation for the purity and composition of APA, STA, and SAA.

The GC-FID analysis for each compound revealed specific retention times, which helped identify the main product and any impurities. For example, APA was analyzed using an Agilent DB-WAX column with helium as the carrier gas, while STA and SAA utilized an Agilent HP-5 column. In this context, the retention time for APA was found to be 17.0 min, which helped distinguish it from potential contaminants. The consistent detection of impurities at levels below 1.5% indicates a high degree of purity, supporting the reliability of the experimental results.

The in vitro tensile strength test is a fundamental method for evaluating the mechanical properties of hair fibers treated with the synthesized compounds [41]. This test measures the force required to extend hair fibers to a specific length, providing insights into the strength and elasticity of the hair [47,48,49]. In this study, the tensile strength tests demonstrated significant improvements in the mechanical properties of hair treated with APA, STA, and SAA compared to untreated samples. In our study, the force required to pull the hair fibers to 15% extension and the e-modulus were measured before and after treatment. The results indicated that APA and SAA showed the highest efficacy in restoring tensile strength and elasticity, highlighting their potential as effective hair repair agents. The decrease in tensile strength parameters post-treatment compared to baseline measurements suggests an improvement in hair integrity. This is particularly important as it directly correlates with the compounds’ ability to repair and strengthen hair by re-establishing disulfide bonds within the keratin structure [24], and therefore its value as an anticipated compound effective for hair repair.

The ability of the thiol-reactive agents to restore disulfide bridges in hair keratin has been tested using the cysteine reactivity test [46,50]. This test involves reacting the synthesized compounds with cysteine, which mimics the natural disulfide bonding process in hair. The successful regeneration of disulfide bonds is a key indicator of the compound’s efficacy in repairing hair damage [37,46]. The findings from the cysteine reactivity tests revealed that APA demonstrated the most promising results, particularly at higher temperatures, indicating its strong potential to restore disulfide bonds effectively. The presence of mono- and bis-adducts, as confirmed by (LC-MS), further validated the compounds’ ability to react with cysteine and form stable disulfide bridges [45]. This capability is crucial for repairing the internal structure of damaged hair, thereby improving its mechanical properties and overall health.

The combined use of HNMR, QNMR, GC-FID, tensile strength tests, and cysteine reactivity tests provides a comprehensive understanding of the synthesized compounds’ effectiveness in hair repair. HNMR and QNMR ensured the structural integrity and purity of the compounds, which is fundamental for their reliable application in further tests. GC-FID added another layer of purity assessment, confirming the compounds were free from significant impurities. The tensile strength tests correlated well with the cysteine reactivity tests, both indicating that APA and SAA are particularly effective in restoring hair strength and elasticity [41]. The significant improvements in mechanical properties observed in tensile strength tests were supported by the successful formation of disulfide bonds in the cysteine reactivity tests. This correlation underscores the compounds’ potential to repair damaged hair by chemically restoring its internal structure.

The control reagent Olaplex^®^ (Olaplex, Inc., New York, NY, USA), as referenced in US Patent 9,597,273 B2 [51], serves as a benchmark for evaluating the efficacy of APA, STA, and SAA. Olaplex^®^ is recognized for its ability to repair disulfide bonds in chemically treated hair, thus reducing damage and breakage. It contains *bis-aminopropyl diglycol dimaleate*, which effectively reconnects broken disulfide bonds in hair keratin [51,52]. Comparing Olaplex^®^ with the synthesized thiol-reactive agents, it is crucial to consider their mechanisms of action. Olaplex^®^ primarily works by forming new disulfide bonds, restoring the hair’s structural integrity and strength [51,52]. APA was confirmed by LC-MS to form both mono- and bis-adducts with cysteine, indicative of its bifunctional thiol-reactivity and potential to generate cross-links between keratin thiol groups. STA and SAA, which lack structural bifunctionality, formed only mono-adducts. These findings support the proposed mechanism by which APA restores tensile strength in bleached hair via chemical bridging of cysteine residues, with cysteine being the main component of keratin and imparting strong covalent bonds and insolubility [53]. This additional pathway potentially offers a more robust repair mechanism. The tensile strength and cysteine reactivity tests highlighted the superior performance of APA and SAA compared to Olaplex^®^. The enhanced tensile strength and effective disulfide bond formation observed with APA and SAA indicate their higher efficacy in repairing and strengthening hair fibers.

Our research work reveals several strengths, including the novelty of the synthesized compounds and their ability to redefine pathways for hair repair. The study employed a robust methodology and well-established in vitro tests, ensuring reliable and reproducible results. Additionally, the use of advanced chromatography techniques provided precise and comparable data on the efficacy of the compounds. However, the study has limitations, as it was conducted on a specific category of artificial hairs, necessitating the inclusion of different hair types to generalize the results. Furthermore, the findings need to be supported by in vivo tests to create a comprehensive outlook for ongoing research and validate the compounds’ effectiveness in real-world applications.

In conclusion, the findings of our study suggest that the synthesized thiol-reactive cross-linking agents have the potential to be optimized into advanced hair repair treatments for the cosmetic industry. The experimental results indicate that the newly developed thiol cross-linking agents hold promise for repairing hair damage by chemically rejuvenating disulfide bridges, presenting a novel and potentially impactful direction for the cosmetic industry.

## 4. Materials and Methods

Many cosmetic products on the market aim to repair, alleviate, or minimize hair damage by restoring disulfide bridges through cross-linking thiol moieties. Many developed thiol-reactive cross-linking agents are capable of cross-linking thiol moieties, thus emulating disulfide bridges. The thiol moieties to be cross-linked may result from prior cleavage of disulfide bridges as a result of physical or chemical hair treatments. By cross-linking thiol moieties, thiol-reactive cross-linking agents enable the restoration of disulfide bridges. These agents can comprise at least two electrophiles that are linked by a fragment comprising at least one C_3–11_ cycloalkyl group. These electrophiles can be selected from a wide variety of chemical reagents, and surprisingly, the addition of at least one cycloalkyl group renders the thiol-reactive cross-linking agents amphiphilic. The C_3–11_ cycloalkyl groups are noninvasive and environmentally benign, reducing toxicity and enhancing safety.

Another group of cross-linking reagents comprises four different families of salts of diverse formulas that can link two thiol moieties.

Based on the above, a series of salts were synthesized and characterized to mimic the repair function that also employs a bicyclic moiety in the core of the linker to bestow the structure with an amphiphilic property, enhance hair volume and slickness, and take advantage of the benign noninvasive properties compared to aromatic compounds in the linker. All methods were performed in accordance with the relevant guidelines and regulations.

The compounds APA, STA, and SAA (Table 3) were synthesized and chemically characterized by SpiroChem AG, a Swiss-based contract research organization (Basel, Switzerland), under the project code internally referred to as ‘Tara Brand’ for confidentiality and traceability within the collaborative framework. The active test compounds (APA, STA, SAA) were evaluated against two references:

(1) An internal benchmark compound provided by Proderm GmbH (composition not publicly disclosed);
(2) The commercially available Olaplex No. 0 Intensive Bond Building Treatment (Olaplex, Inc., New York, NY, USA), which contains bis-aminopropyl diglycol dimaleate as its active ingredient [51,52].

**Table 3:** Code and structures of compound APA.

Chemical Structure	Code
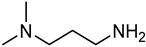	APA-1
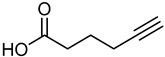	APA-2
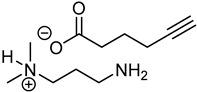	APA

### 4.1. Synthesis and Characterization of 3-Amino-N,N-dimethylpropan-1-aminium Hex-5-ynoate (APA)

The three thiol-reactive salts APA, STA, and SAA were synthesized and fully characterized using nuclear magnetic resonance spectroscopy (^1^H-NMR, ^13^C-NMR), quantitative NMR (qNMR), and gas chromatography–flame ionization detection (GC-FID). The results of these analytical techniques confirmed structural identity, purity, and reproducibility across synthesized batches. Detailed characterization is presented alongside the synthetic route of each compound below. The preparation process was successfully developed, and a 50% (*w*/*w*) aqueous solution of APA was obtained, corresponding to 50 g of active compound per 100 g of total solution.

#### 4.1.1. Materials for APA

APA-1 was purchased from Shanghai Titan Technology Company Limited, whereas APA-2 was purchased from Shanghai Youde Agricultural Technology Company Limited. All compounds were received as analytically verified aqueous solutions with GC-FID-confirmed purities of ≥97.9% and quantified by qNMR for active content. No additional purification was necessary post-delivery, as all batches met the acceptance criteria for in vitro formulation and testing.

#### 4.1.2. Preparation of APA

Under a positive stream of dry nitrogen (≥99.999%, 30–50 mL/min, ~0.1–0.2 bar overpressure), APA-2 was charged into a jacketed borosilicate glass reactor (Reactor R1, 3 L working volume), equipped with a four-neck lid, overhead mechanical stirrer, and external temperature control (maintained at 20–25 °C). Then, 500 mL of H_2_O was charged into reactor R1 in the same temperature range. The APA-1 aqueous solution (50% *w*/*w*) was added dropwise into Reactor R1 via a PTFE stopcock-equipped addition funnel under continuous stirring (250–350 rpm) using an overhead mechanical paddle stirrer. The reactor was maintained at −5 to +5 °C with a recirculating chiller and nitrogen atmosphere to ensure homogeneous mixing and suppress side reactions. After addition, the batch was stirred for 3 h at 5–10 °C. A small-scale compatibility test was conducted by mixing APA-1 and APA-2 solutions in a 1 L four-neck borosilicate glass reactor at 5 °C. The test confirmed homogeneity and chemical stability, validating the conditions for subsequent full-batch synthesis. After passing the test, the sample was put into a four-neck bottle and kept sealed.

### 4.2. Synthesis and Characterization of Succinic Acid (N,N,N-triallylamine) Salt (STA)

It was necessary to develop and manufacture 1.0 × 10^3^ g of *succinic acid (N,N,N-triallylamine) salt* (STA) (Table 4). The preparation process was successfully developed by manufacturing a batch of 40% STA aqueous solution, and 2.61 × 10^3^ g of qualified 40% STA aqueous solution was obtained. STA synthesis was initiated using commercially available triallylamine (≥98%) and succinic acid (≥99%), each verified by GC-FID and HPLC, respectively. No additional purification was required prior to use. The reaction proceeded in MeCN with subsequent isolation of the STA salt as a 40% *w*/*w* aqueous solution.

#### 4.2.1. Synthesis of STA

STA-1 and STA-2 were purchased from Shanghai Titan Technology Company Limited.

#### 4.2.2. Preparation of STA

Under nitrogen, 5.1 × 10^2^ g of STA-1 was charged into reactor R1 at 20–25 °C, and then H_2_O (7.5 × 10^2^ g) was charged into reactor R1 at 20–25 °C. The next step was to prepare a NaOH solution in which 1.8 × 10^2^ g of NaOH was dissolved in 2.5 × 10^2^ g of water. Afterward, for the configuration of the STA-2 HCl solution, under nitrogen, 6.5 × 10^2^ g of STA-2 was charged into reactor R2. The reaction was carried out in Reactor R2, a 3 L jacketed borosilicate glass vessel equipped with a four-neck lid, mechanical stirring (250–350 rpm), and inert gas protection. Aqueous HCl (37% *w*/*w*, approx. 12 M) was added dropwise until the reaction mixture reached pH 3.5–4.0, enabling complete salt formation. HCl solution containing concentrated hydrochloric acid was then slowly added at 10–20 °C. The reaction took place at a controlled T = 10–20 °C, and then the NaOH solution was added to R1. After addition, the batch was stirred for 2 h at 10–20 °C. Afterward, the STA-2 HCl solution was slowly added to R1 at 10– 20°C and then stirred for 2–4 h at 10–20 °C. Temperature was controlled via a double-jacketed glass reactor connected to a recirculating thermostatic bath (±1 °C accuracy), maintaining the reaction mixture at 20–25 °C throughout the synthesis process. A preliminary solubility and compatibility test was performed on a small scale, in which triallylamine and succinic acid were combined in an aqueous medium at room temperature. The resulting STA salt solution was homogenous and stable, confirming suitability for scale-up under the intended synthesis conditions. After passing the test, the solution was put into a Teflon bottle and kept sealed. STA-1 and STA-2 must be pretreated with NaOH and HCl. The pretreated solution must be clarified before mixing. If needed, the mixture can be heated to 50 °C until clarified.

The proposed process is described below after summarizing the previous experiments. The dosage ratio of STA-1 to STA-2 is 1:1.05. The STA-1 and STA-2 have been premixed with NaOH and HCl, and the two solutions must be clarified after mixing. The solvent was water (2 V).

### 4.3. Synthesis and Characterization of the Succinic Acid Allylamine Salt (SAA)

#### 4.3.1. Manufacturing Process of SAA

SAA-1 and SAA-2 were purchased from Shanghai Titan Technology Company Limited. Commercially available allylamine (≥98%) and succinic acid (≥99%) were used without further purification after verification by GC-FID and HPLC, respectively (Table 5). Allylamine was handled under an inert atmosphere to prevent volatilization, and succinic acid was pre-dissolved in water with pH adjustment using NaOH prior to combination.

#### 4.3.2. Preparation of SAA

Under nitrogen, 1.03 × 10^3^ g of SAA-2 was charged into reactor R1 at 20–25 °C. The next step was to prepare a NaOH solution in which 2.62 × 10^2^ g of NaOH was dissolved in 4.2 × 10^2^ g of water. The reaction proceeded at a controlled T of 10–20 °C, after which NaOH solution was added to R1 (solution 1). After the addition, the mixture was stirred for 1 h at 10–20 °C. In reactor R2, SAA-1 (705 g) was mixed with 1.0 × 10^3^ g of water, and then solution 1 was slowly added to R2 and stirred for 2–3 h at 20~25 °C. A small-scale compatibility and solubility test was conducted by combining allylamine and succinic acid in aqueous medium at room temperature. The clear and stable salt solution formed without phase separation, supporting its readiness for scale-up. After passing the test, the solution was put into a four-neck bottle and kept sealed.

### 4.4. Identification of APA

To identify the final product, ^1^H-NMR spectroscopy was used. The solvent used for identification was methanol-d_4_ (CD_3_OD). All NMR spectra were recorded using a Bruker Avance III HD 400 MHz spectrometer (9.4 Tesla) equipped with a BBO probe. Samples were maintained at 298 K (25 °C) throughout acquisition. Spectra were processed using Bruker TopSpin software (Bruker BioSpin, Billerica, MA, USA, available at https://www.bruker.com/en/), and quantitative analysis was performed using certified internal standards.

### 4.5. QNMR

Quantitative ^1^H-NMR (qNMR) was performed using certified internal standards (1,3,5-trimethoxybenzene for APA; pyrazine for STA and SAA). Signals were integrated using Bruker TopSpin (Bruker BioSpin, Billerica, MA, USA, available at https://www.bruker.com/en/), and calibration was based on the known amount and proton count of the internal standard. Analyte signals were selected for their clarity and non-overlapping nature, and concentration was calculated using molar ratio equations. All assays were performed in duplicate, yielding relative standard deviations below 2%. Structural verification was performed using ^1^H-NMR and qNMR as primary techniques, complemented by ^13^C-NMR and high-resolution mass spectrometry (HRMS) to provide orthogonal confirmation of molecular structure and molecular weight. While X-ray crystallography was not feasible due to the non-crystalline nature of the salts, spectral data across methods confirmed consistency with the proposed structures.

### 4.6. Assay: (QNMR, %w/w)

Residual solvent analysis was conducted using an internal GC-FID method (GP-QC011), validated in accordance with ICH Q3C guidelines. This method is comparable to established procedures such as those described by Hassan et al. (2021) [54] for the determination of organic solvents in pharmaceutical samples. The GP-QC011 method was adopted, using a BRUKER AV400 Nuclear Magnetic Resonance spectrometer. All samples and internal standards were weighed using a calibrated analytical microbalance with a precision of 0.01 mg (Mettler Toledo XPR205D, Mettler-Toledo GmbH, Greifensee, Switzerland), ensuring accurate mass determination for qNMR and GC-FID assays. The reagents included deuterated methanol and 1,3,5-trimethoxybenzene (internal standard), with CD_3_OD as the solvent. Approximately 0.02 to 0.03 g of sample was added to a sample tube, 0.02–0.03 g of 1,3,5-trimethoxybenzene was added, 0.6 mL of CD_3_OD was added to dissolve the sample, and the sample was subsequently transferred to a nuclear magnetic resonance tube. Chemical shifts were referenced to the residual proton signals of the deuterated solvents used: HOD at 4.79 ppm in D_2_O and CHD_2_OD at 3.31 ppm in CD_3_OD for ^1^H-NMR, and 49.0 ppm for CD_3_OD in ^13^C-NMR. All shifts are reported in ppm (δ). Impurity signals in ^1^H-NMR spectra were minimal, representing <1.3% of the total integral for APA and STA, and <1.5% for SAA. These levels were consistent with GC-FID and qNMR assessments, confirming high chemical purity suitable for structural and quantitative analysis. The content was calculated according to the following formula:

$$Assay\%=\frac{A_{sample}\times W_{ISTD}\times MW_{sample}\times N_{ISTD}\times P_{ISTD}}{A_{ISTD}\times W_{sample}\times MW_{ISTD}\times N_{sample}}$$

where

A_sample_: Represents the integral value of the sample signal;

A_ISTD_: represents the integral value of the internal standard signal;

W_ISTD_: The weight of the sample (g);

W_sample_: represents the weight of the internal standard (g);

P_ISTD_: The percentage of weight of the internal standard (%);

N_sample_: represents the reference proton number of the internal standard;

N_ISTD_: Represents the reference proton number of the sample;

MW_ISTD_: Molecular weight of internal standard;

MW_sample_: represents the molecular weight of the sample.

### 4.7. Identification of STA

To identify the final product, ^1^H-NMR spectra were recorded at 400 MHz (9.4 T) using a Bruker Avance III HD spectrometer at 298 K (25 °C). The chemical shift scale was referenced to the residual solvent peaks: HOD at 4.79 ppm (D_2_O) and CHD_2_OD at 3.31 ppm (CD_3_OD)**.** The solvent used for identification was water-d_2_ (D_2_O). For sample solution preparation, 0.02–0.03 g of sample was removed from the sample tube, 0.5–1.0 mL of D_2_O was added to dissolve the sample, and the sample was subsequently transferred to a nuclear magnetic resonance tube.

QNMR was used to quantify the products and impurities present. The analysis was conducted using Method No. GP-QC011. The instruments included a BRUKER AV400 NMR and an electronic balance. Deuterated methanol pyrazine was used as the internal standard reagent, and D2O was used as the solvent. Approximately 0.03–0.035 g of sample was added to a sample tube, 0.02–0.03 g of pyrazine was added, 0.6 mL of D_2_O was added to dissolve the sample, and the sample was subsequently transferred to a nuclear magnetic resonance tube. The proton peak with a chemical shift of 2.5 ppm was taken as the sample signal integral, and the proton peak with a chemical shift of 8.5 ppm was taken as the internal standard signal integral. The content was calculated according to the same formula used for APA analysis.

### 4.8. Identification of SAA

To identify the final product, HNMR spectroscopy was used. The solvent used for identification was water-d_2_ (D_2_O). For sample solution preparation, 0.02–0.03 g of sample was removed from the sample tube, 0.5–1.0 mL of D_2_O was added to dissolve the sample, and the sample was subsequently transferred to a nuclear magnetic resonance tube.

Quantitative nuclear magnetic resonance (QNMR) was employed to quantify the products and impurities present. The analysis followed Method No. GP-QC011. The equipment included a BRUKER AV400 NMR and an electronic balance. Deuterated methane served as the internal standard reagent, with D_2_O as the solvent. For the sample solution, approximately 0.025–0.03 g of the sample was added to a sample tube, along with 0.02–0.03 g of pyrazine. Then, 0.6 mL of D_2_O was added to dissolve the sample, which was subsequently transferred to a nuclear magnetic resonance tube. The proton peak at a chemical shift of 2.5 ppm was used as the sample signal integral, and the proton peak at 8.5 ppm was used as the internal standard signal integral. The content was calculated according to the formula used in the previous two compounds.

### 4.9. Specification and Analytical Method of the Final Product

#### 4.9.1. Gas Chromatography—Flame Ionization Detector (GC–FID) Analysis of APA

GC-FID analyses of APA, STA, and SAA were performed using the same chromatographic method, differing only in sample dilution solvent. Each salt was diluted to 1 mg/mL and injected using a DB-200 capillary column under identical temperature programming and detector settings. Chromatographic purity exceeded 97.9% for all compounds, with low levels of total impurities (<1.5%) and no significant byproducts. The product to be tested was APA, which was originally prepared. The diluent used was acetonitrile (ACN). The blank used was the diluent as well. The sample solution to be tested was 0.06 g/mL, which was prepared by weighing 0.06 g into a sample bottle. Then, 1 mL of the diluent was added to dissolve the product, and the solution was mixed well. Samples were stored in amber-colored, pharmaceutical-grade HDPE bottles with PTFE-lined screw caps to ensure chemical stability and minimize light, air, and moisture exposure. Each sample was prepared and analyzed in duplicate to ensure reproducibility and confirm the consistency of GC-FID measurements. Each sample (1 mg/mL) was injected in duplicate using a 1.0 µL injection volume under a split ratio of 10:1. The room temperature was stable for 24 h.

Purity was calculated using the normalized peak area method, excluding solvent peaks. This approach was selected due to the absence of certified external standards for the test compounds and the high purity/simplicity of the sample matrix, allowing accurate peak separation and integration. The results were consistent across duplicates and aligned with independent qNMR data. The retention time for the substance APA was 17.0 min. The specifications of the GC-FID are described as follows. The column used was Agilent DB-WAX, 30 m × 0.25 mm × 0.25 µm, PN: 122-7032, with Helium as the carrier gas at a flow of 1.0 mL/min. The inlet temperature was set at 260 °C with a split ratio of 50:1. The oven temperature was set at 50 °C with a hold time of 2 min, then 150 °C and 250 °C with a hold time of 1 min and 8 min, respectively. The FID detector temperature was set at 300 °C with a hydrogen flow rate of 40 (mL/min), an air flow of 400 (mL/min), an auxiliary flow rate (He) of 25 (mL/min), and a run time of 25 min. The needle wash solvent was ACN with an injection quantity of 1 µL.

#### 4.9.2. GC–FID Analysis of STA

To detect the presence of related substances or impurities, samples were subjected to GC-FID according to the following specifications. The product to be tested was STA, which was originally prepared. The diluent used was methanol (MeOH). The blank used was the diluent as well. The sample solution to be tested was 0.02 g/mL, which was prepared by weighing 0.02 g into a sample bottle. Then, 1 mL of the diluent was added to dissolve the product, which was mixed well. Two parallel copies, S1 and S2, were prepared. The injection times of sample solution S1 and sample solution S2 were 1. The room temperature was stable for 24 h.

The result calculation was based on area normalization (regarding the peaks shown in the blank), and impurity peaks ≥0.05% were reported. The retention time for the substance STA-1 was 15.521 min, and that of STA-2 was 17.415 min. The specifications of the GC-FID are described as follows. The column used was Agilent HP-5, 30 m × 0.53 mm × 3 µm, PN:19095J-623, Helium as carrier gas with a flow rate of 5.58 mL/min. The inlet temperature was set at 250 °C with a split ratio of 10:1. The oven temperature was set at 40 °C with a hold time of 5 min, then 160 °C and 250 °C with a hold time of 0 min and 5 min, respectively. The FID detector temperature was set at 260 °C with a hydrogen flow rate of 40 (mL/min), an air flow of 400 (mL/min), an auxiliary flow rate (N_2_) of 30 (mL/min), and a run time of 25 min. The needle wash solvent was MeOH with an injection quantity of 1 µL.

#### 4.9.3. GC–FID Analysis of SAA

To detect the presence of related substances or impurities, samples were subjected to GC-FID according to the following specifications. The product to be tested was SAA, which was originally prepared. The diluent used was MeOH:H_2_O at a ratio of 1:1. The blank used was the diluent as well. The sample solution to be tested was 0.05 g/mL, which was prepared by weighing 0.05 g into a sample bottle. Then, 1 mL of the diluent was added to dissolve the product, and the solution was mixed well. Two parallel copies, S1 and S2, were prepared, and the injection times of sample solution S1 and sample solution S2 were 1. The room temperature was stable for 24 h.

The result calculation was based on area normalization (regarding the peaks shown in the blank), and impurity peaks ≥0.05% were reported. The retention time for SAA-1 was 19.007 min, and that for SAA-2 was 4.369 min. The specifications of the GC-FID are described as follows. The column used was Agilent HP-5, 30 m × 0.53 mm × 3 µm, PN:19095J-623, with Helium as carrier gas, with a flow rate of 5.58 mL/min. The inlet temperature was set at 250 °C and a split ratio of 10:1. The oven temperature was set at 40 °C with a hold time of 5 min, then 160 °C and 250 °C with a hold time of 0 min and 5 min, respectively. The FID detector temperature was set at 260 °C with a hydrogen flow rate of 40 (mL/min), an air flow of 400 (mL/min), an auxiliary flow rate (N2) of 30 (mL/min), and a run time of 25 min. The needle wash solvent was MeOH:H_2_O = 1:1 with an injection quantity of 1 µL.

### 4.10. Tensile Strength Test

The in vitro method for investigating the tensile strength of human hair fibers was designed to determine the effects of hair strengthening or protection on hair care and/or hair cleansing products. In this study, the force needed to pull single hair fibers until 15% extension, as well as the e-modulus in the resulting force curve, were determined as the main parameters. The values after application of the test products were compared to the baseline values before application. The test products were divided into two groups: Group 1 included test products at different concentrations of an active substance and the respective reference compound, and Group 2 included test products at different concentrations of the active substance in a different base and the respective Olaplex 0 reference. The commercially available Olaplex No. 0 Intensive Bond Building Treatment (Lot # 2023-04B-O0) was used as a benchmark (Reference Sample K). This product contains bis-aminopropyl diglycol dimaleate and was applied at a concentration of 2.5% in accordance with ProDERM’s standardized protocol.

## 5. Method

The test was conducted in accordance with the study protocol [48] and according to the principles of the quality standard ISO 9001 [55] to meet customer and regulatory requirements.

### 5.1. Hair Tresses

Tensile strength testing was conducted using untreated virgin Caucasian hair strands (light to medium brown, Level 5–6), 20–25 cm in length and 60–80 µm in diameter, provided under standardized sourcing by ProDERM (GmbH, Schenefeld, Hamburg, Germany). Hair strands were pre-damaged using a standardized bleaching procedure involving application of a commercial bleach mixture (6% hydrogen peroxide with ammonium persulfate) for 45 min at room temperature. The strands were rinsed, shampooed, and air-dried without further treatment to simulate oxidative hair damage prior to tensile strength testing. Fifty fibers of natural hair (European) were examined for each treatment.

### 5.2. Climatic Conditions

Tensile strength measurements were performed in a climate-controlled facility (20 ± 2 °C; 65 ± 2% RH), in accordance with ISO 139:2005. Although samples were conditioned within a 40–70% RH range prior to testing, all mechanical assays were conducted under tightly regulated humidity conditions to minimize the effect of moisture variability on tensile properties.

### 5.3. Test Procedure

#### 5.3.1. Instrumental Measurements (t_0_): Baseline Before Treatment

Fifty fibers per product were prepared by crimping the fibers at a defined hair length. Hair fiber diameters were measured using a non-contact laser scan micrometer (Keyence LS-7030M, resolution 0.01 µm), with three replicates per strand to obtain an average diameter value.

Hair strands were allowed to air dry at 22 ± 2 °C and ambient humidity (45–55%) for at least 4 h in a dust-free, ventilated environment prior to mechanical testing. The hair fibers were conditioned overnight under the climatic conditions mentioned below. Afterward, the product treatment was performed on 38 fibers per product as follows: Each hair strand was secured in a custom-built, rubber-padded polyoxymethylene (POM) holder that maintained a 5 cm gauge length without inducing mechanical stress. Test formulations were applied for 2 min at 22 ± 1 °C, after which the strands were rinsed and prepared for tensile testing. Following rinsing, all hair strands were conditioned overnight at 22 ± 2 °C and 65 ± 2% relative humidity, suspended freely in a ventilated and dust-free environment to allow uniform moisture equilibration.

#### 5.3.2. Instrumental Measurements (t1): After Treatment

The cross-sectional area of each fiber was measured by laser measurements using the FDAS675 unit (Dia-Stron, Hampshire, UK), which is a laser scanning system for rapid analysis of the cross-sectional data from hair (or other small fibers). Then, the tensile measurements were repeated.

### 5.4. Statistical Analysis

The analysis was performed for pairs of Group 1 substances compared to the reference Group 1 substances and Group 2 substances compared to the reference Group 2 or Olaplex substances. The raw data of all the valid hair samples were listed by treatment and assessment time. Differences relative to baseline were computed as (ti − t0)/t0 × 100% and are listed by treatment and assessment time, where it was any assessment time after baseline t0. N, mean, standard deviation, median, minimum, maximum, and box plots are presented for the raw data and differences relative to the baseline for each parameter according to treatment and assessment time. A significance level of 0.05 (alpha) was chosen for statistical analysis. Due to the explorative character of the study, no adjustments for multiplicity were made. For each parameter, comparisons against the 0% benchmark were performed on relative differences to baseline by the Wilcoxon signed rank test. Pairwise comparisons of treatments within each group of products were performed on relative differences to baseline by the Mann–Whitney–Wilcoxon test. Tensile data were collected and analyzed using ZwickRoell testXpert III software, Version 1.6 (build 1.6.0.553), configured for constant-rate tensile testing at 10 mm/min, with automatic determination of mechanical parameters.

### 5.5. Cysteine Reactivity Test

Cysteine and cystine are both crystalline amino acids. Cystine occurs in keratin proteins in hair and yields cysteine when reduced. When chemical treatments are used on hair, cystine reverts to cysteine, weakening the strands. Hair breaks down with repeated chemical treatments; therefore, by restoring the hair to its natural cystine state, the damage is repaired [38,56]. The aim of this experiment was to test cysteine reactivity with APA, STA, and SAA. N-Cbz-L-cystine (≥98%, Bachem AG, Cat. No. 4010126) was used as the model substrate for thiol recombination studies. All solvents and buffers were analytical or LC-MS grade. Adduct formation was monitored by LC-MS, with APA achieving up to 81.5% bis-adduct formation at 50 °C. No further purification of reaction mixtures was performed, and product purity was estimated by area normalization (mono- and bis-adducts ≥85–90%). N-Cbz-L-cystine was purchased from Bachem AG (≥98%, Cat. No. 4010126) with verified HPLC purity. While NMR characterization was not repeated, spectra provided by the supplier are consistent with published ^1^H-NMR data. Cysteine reactivity was tested while using APA/STA/SAA as the active ingredient. In the next assay, different batches of APA/STA/SAA were prepared. The reaction proceeded to full conversion with 100% theoretical yield based on mass recovery, and the resulting aqueous APA solution contained ~52.3% active ingredient as determined by quantitative ^1^H-NMR (qNMR). In the second batch, 126 g of STA aq. was obtained with 100% yield and ~45.6% yield; HNMR confirmed that the obtained product was desired. For the third batch, 116 g of SAA aq. was obtained in a ~38.8% reaction; HNMR confirmed that the obtained product was desired. In parallel, 5.00 g of N-Cbz-L-cystine was reduced to N-Cbz-l-cysteine (3.0 g) with PPh3 in THF. Adduct formation was confirmed by LC-MS, where the reactivity of APA, STA, and SAA toward N-Cbz-L-cystine was quantified under buffered, physiologically relevant conditions. LCMS traces of selected cysteine tests in pH = 5.4 buffer solutions were then performed on APA/STA/SAA at 30 °C and then at 50 °C.

### 5.6. Batch Process Development and Optimization

#### 5.6.1. Batch Process Development and Optimization for APA

Two batches of APA were prepared, APA-20230090-E0001 and APA-20230090-E0002. APA-1 and APA-2 were diluted with purified water, and then the aqueous solution of APA-1 was slowly added to the reaction bottle at −5–5 °C. Next, the temperature was increased to 5–10 °C, and the mixture was stirred for 2–3 h. Thin layer chromatography (TLC) was utilized as a rapid, qualitative analytical technique to monitor reaction progress during the synthesis of APA, STA, and SAA. Specifically, it was employed to track the disappearance of starting materials (e.g., amines or acids) and to detect the presence of byproducts or unreacted components during reaction optimization. TLC analyses were conducted using silica gel 60 F_254_ pre-coated aluminum sheets (Merck; 0.25 mm thickness). The mobile phase used for APA development consisted of chloroform/methanol/water (65:25:4, *v*/*v*/*v*), while for STA and SAA, a system of ethyl acetate/acetic acid/water (60:20:20, *v*/*v*/*v*) was employed. Samples (~1 µL) were applied using calibrated glass capillaries. Plate development was carried out at room temperature (22 ± 1 °C) after a 15-min chamber saturation period. The development distance was standardized to approximately 7.5 cm across all experiments. Detection of compounds was performed under UV light at 254 nm to visualize Cbz-protected and aromatic species. Ninhydrin staining, followed by heating at 100 °C for 3 min, was used to identify free amines. Additionally, KMnO_4_ staining was applied to detect polar, non-UV-absorbing compounds. Rf values were calculated and monitored throughout the synthesis process, with product bands compared to reference standards when available to confirm identity and assess purity. The structure of the product was verified by HNMR. The output for both batches was an aqueous yellowish solution.

#### 5.6.2. Batch Process Development and Optimization for STA

A total of 16 batches of the salt STA were prepared under different conditions but with the same IPC. All samples were analyzed by HNMR. The output was yellow oil in 8 of the 16 samples, Tawny oil in 2 of the samples, while the rest was a light yellow solution of GC purity of 99.7%. Ten early-stage batches (5 APA, 3 STA, 2 SAA) were deemed unsuccessful due to issues such as incomplete conversion, phase separation, or improper pH control during salt formation. These batches were excluded from all testing. The final synthesis protocols were developed based on corrective optimizations applied in response to these failures.

#### 5.6.3. Batch Process Development and Optimization for SAA

A total of 3 batches of the salt SAA were prepared under different conditions, but with the same IPC. The samples were analyzed by HNMR. The output was white solid in one of the batches and 2 aqueous colorless liquids in the other batches, with a GC purity of 97.9%.

## Figures and Tables

**Table 1:** Synthesis of STA.

Step	Reagent/Action	Quantity/Conditions	Notes
1	Succinic acid dissolved in water	4.2 g in 50 mL distilled water	Stirred until fully dissolved
2	pH adjustment with NaOH	~1.5 mL of 10 M NaOH added dropwise to pH 5.0–5.5	Ensures solubility and controlled neutralization
3	Addition of triallylamine	5.0 g added dropwise over 10 min under stirring	Exothermic; added at 20–25 °C
4	Acidification with HCl	3.0 mL of 37% HCl (12 M), added dropwise to pH 3.5–4.0	pH monitored in real time
5	Stirring and homogenization	Stirred for 30 min at 22 ± 1 °C	Transparent solution formed
6	Final dilution	Brought to 100 mL with distilled water	Produces 40% *w*/*w* aqueous STA solution
7	QC sampling	TLC and pH analysis performed	Confirmed single spot on TLC and pH ~3.7
8	Storage	Amber HDPE bottle with PTFE-lined cap at room temperature	Chemically stable for 4 weeks

**Table 2:** Cysteine reactivity test.

Agent	APA	STA	SAA
30 °C (Mono-Adduct) 30 min	Present	Present	Not Detected
40 °C (Mono-Adduct) 1 h	Not Tested	Not Tested	Detected
50 °C (Mono/Bis-Adduct) 1 h	12% Mono/Bis	No Bis Detected	No Bis Detected
Second Assay (Mono/Bis-Adducts)	Present	Present	Mono Detected
N-Cbz-L-cysteine 4.0 eq, pH = 5.4, 30 °C, 0.5 h (Mono/Bis)	88.86%/0%	41.03%/0%	Not Detected
N-Cbz-L-cysteine 4.0 eq, pH = 5.4, 30 °C, 1 h (Mono/Bis)	87.57%/0%	47.24%/0%	Not Detected
N-Cbz-L-cysteine 4.0 eq, pH = 5.4, 40 °C, 1 h (Mono/Bis)	Not Tested	Not Tested	26.99%/0%
N-Cbz-L-cysteine 4.0 eq, pH = 5.4, 50 °C, 0.5 h (Mono/Bis)	17.41%/76.72%	96.81%/0%	38.55%/0%
N-Cbz-L-cysteine 4.0 eq, pH = 5.4, 50 °C, 1 h (Mono/Bis)	11.99%/81.54%	97.79%/0%	73.20%/0%

**Table 4:** Codes and structures of compound STA.

Chemical Structure	Code
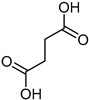	STA-1
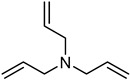	STA-2
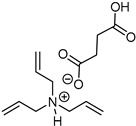	STA

**Table 5:** Codes and structures of compound SAA.

Chemical Structure	Code
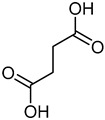	SAA-1
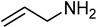	SAA-2
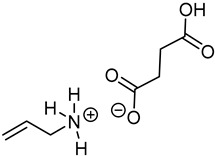	SAA

## Data Availability

The datasets used in the current study are available from the corresponding author (BA) upon reasonable request.
